# Obesity-Induced Methylation of Osteopontin Contributes to Adipogenic Differentiation of Adipose-Derived Mesenchymal Stem Cells

**DOI:** 10.1155/2019/1238153

**Published:** 2019-02-17

**Authors:** Min Tang, Rui Chen, Hao Wang, Guowei Sun, Fan Yin, Beibei Liang, Yang Yang, Gaowa Sharen, Huafeng Wei, Xuyu Zhou, Gang Huang, Jian Zhao

**Affiliations:** ^1^Shanghai Key Laboratory for Molecular Imaging, Shanghai University of Medicine & Health Sciences, Shanghai 201318, China; ^2^Division of Gastroenterology and Hepatology, Institution of Digestive Diseases, Tongji Hospital, Tongji University School of Medicine, Shanghai 200065, China; ^3^Angecon Biotechnology Limited, Shanghai 201318, China; ^4^Air Force Hospital, Northern Theater Command, PLA, China; ^5^The Arctic Temple Clinic, Beijing Fourth Service Center, 8 Garden East Road, Beijing 100191, China; ^6^Cancer Center Key Lab, PLA General Hospital, Beijing 100853, China; ^7^Central Research Institute of Shanghai Pharmaceutical (Group) Co., Ltd., Shanghai 201203, China; ^8^Changhai Hospital, The Second Military Medical University, Shanghai 200438, China

## Abstract

Obesity is a major risk factor for many chronic diseases, including diabetes, fatty livers, and cancer. Expansion of the adipose mass has been shown to be related to adipogenic differentiation of adipose-derived mesenchymal stem cells (ASCs). However, the underlying mechanism of this effect has yet to be elucidated. We found that osteopontin (OPN) is downregulated in ASCs and adipose tissues of obese mice and overweight human beings because of methylation on its promoter, indicating that OPN may affect the development of obesity. Silencing of OPN in wild-type ASCs promotes adipogenic differentiation, while reexpression of OPN reduced adipogenic differentiation in OPN^−/−^ ASCs. The role of extracellular OPN in ASC differentiation was further demonstrated by supplementation and neutralization of OPN. Additionally, OPN suppresses adipogenic differentiation in ASCs through the C/EBP pathways. Consistent with these *in vitro* results, by intravenous injection of OPN-expressing adenovirus to the mice, we found OPN can delay the development of obesity and improve insulin sensitivity. Therefore, our study demonstrates an important role of OPN in regulating the development of obesity, indicating OPN might be a novel target to attenuate obesity and its complications.

## 1. Introduction

Prevalence of obesity and obesity-associated metabolic problems has become a major economic and medical burden worldwide [1, 2]. Obesity is a key risk factor for the development of type 2 diabetes mellitus (T2DM), cancer, and cardiovascular disease [3]. By 2030, nearly 60% of the world's adult population might be either overweight or obese [4]. White adipose tissue (WAT), the major type of adipose tissues, functions as a storage depot of lipids [5, 6]. WAT is also an endocrine organ secreting adipokines which plays key roles in the pathogenesis of obesity and its complications [7, 8]. In obese individuals, the adipose tissue constitutes almost half the body weight, making it the largest endocrine organ in human beings. Even minor metabolic changes in such a large secretory organ have the potential to affect broadly the entire body [9]. Therefore, a better understanding of adipose tissue biology is crucial to design targeted interventions which can attenuate obesity and minimize its deleterious effects.

It has been reported that adipose-derived mesenchymal stem cells (ASCs) play an important role in the development of obesity [9]. ASCs are an important adipose-resident population, which maintain adipose tissue homeostasis through regulation of the quantity of mature adipocytes [10]. Over the past decades, ASCs have been used in clinical therapy protocols in a broad range of diseases [11], due to their potent immunomodulatory properties [12, 13] and their intrinsic ability to differentiate into multiple cell lineages, such as adipocytes, osteoblasts, chondrocytes, and neurons [14, 15]. ASCs, under defined conditions *in vitro*, can differentiate into adipocytes depending on the presence of adipogenic factors (e.g., isobutylmethylxanthine, indomethacin, and dexamethasone) [14, 16]. It has been shown that such adipogenic factors can activate transcription factor C/EBPs, which are critical for adipogenesis, the process whereby undifferentiated progenitor cells differentiate into adipocytes [9, 17].

Osteopontin (OPN), an arginine-glycine-aspartate-containing glycoprotein, is known as a soluble protein that is present in most body fluids. As an important inflammatory cytokine originally identified in osteoblasts, OPN has multiroles in bone remodeling, cell migration, immune regulation, and tumor metastasis [18, 19]. OPN exists in both secreted and intracellular isoforms, and previous studies showed that sOPN and iOPN have diverse functions [20]. Recently, it was reported that OPN regulates the differentiation of mesenchymal stem cells (MSCs) through its interactions with integrins, expressed cell surface receptors [21]. MSCs can be derived and propagated *in vitro* from different organs and tissues, such as bone marrow, embryos, and adipose tissues. ASCs are the kind of MSCs derived from adipose tissues [22].

However, the mechanisms by which OPN regulates adipogenesis by ASCs during the development of obesity remain poorly understood. Therefore, the aim of this study was to determine the role of OPN in adipogenic differentiation by ASCs during the process of obesity.

## 2. Materials and Methods

### 2.1. Animal Studies and Diets

C57BL/6J mice (wild type (WT)) were purchased from the Shanghai Experimental Animal Center of Chinese Academic of Sciences (Shanghai, China) (http://www.slaccas.com). OPN^−/−^ C57BL/6 mice (B6.Cg-Spp1tm1blh/J, cat. no. 004936) were obtained from Jackson Laboratory (CA, USA) (http://www.jax.org). In the construction of the obese mice experiment, C57BL/6 (male, 8 weeks old) mice were randomly assigned to normal chow diet (CD) (20% kJ/fat, 60% kJ/carbohydrate, and 20% kJ/protein; Trophic High-Tech, Jiangsu, China) groups and high-fat diet (HFD) (60% kJ/fat, 20% kJ/carbohydrate, and 20% kJ/protein; Trophic High-Tech, Jiangsu, China) groups. Each cage housed 4 mice for 12 weeks. *In vivo* OPN effect verification experiment, C57BL/6 (male, 8 weeks old) mice were randomly assigned to two 60% kcal high-fat (60 kcal percent fat, 20% kJ/carbohydrate, and 20% kJ/protein; Trophic High-Tech, Jiangsu, China) groups. Each cage housed 4 mice which received either adenovirus vehicle (pcmv) or 10^8^ PFU once a week of adenovirus OPN for 8 weeks (intravenous injection). Food consumption was determined by measuring the difference between the amount provided and the amount left every three days. Food intake per mouse was calculated based on the amount consumed divided by time and the number of mice per cage. The body weight of each mouse was measured on an electronic balance once per week. All animals were kept and bred in environmentally controlled and specific pathogen-free conditions in the Animal Unit of Shanghai Second Military Medical University of the People's Republic of China. All animal experimental protocols were approved in accordance with the guidelines of the Animal Experiment Committee of Shanghai Second Military Medical University of the People's Republic of China.

### 2.2. Study Participants

A total of 8 men aged 25–45 years were enrolled from individuals who visited Changhai Hospital (Shanghai, China). In our study, all participants were then stratified according to body mass index (BMI) into the following two groups: overweight people and normal people. Overweight was defined as 25 ≤ BMI< 30 kg/m^2^ according to the World Health Organization (World Health Organization, 2000). The study protocol was obtained from surgical resections of patients in Changhai Hospital (Shanghai China). Patients' consent and approval were obtained to use these clinical materials for research purposes.

### 2.3. ASC Collection, Culture, and Differentiation

ASCs were obtained from subcutaneous adipose tissue. The inguinal fat pads were harvested and extensively washed with PBS three times. They were then finely minced and followed by digestion with 0.1% collagenase I (Gibco) for 1 h at 37°C in a 25cm^2^ cell culture dish. Next, the cells were filtered through a 70 mm nylon mesh and cultured in low glutamine Dulbecco's modified Eagle's medium (DMEM) supplemented with 10% fetal bovine serum (FBS), 100 U/ml penicillin, and 100 mg/ml streptomycin (complete medium, all from Gibco, Australia, http://www.thermofish.com). The cultures were incubated in an atmosphere of 5% CO_2_ and 37°C. All nonadherent cells were removed after 24 h. Medium was changed every 3 days.

Differentiation of ASCs was induced according to established protocols [21] with minor modification. For adipocyte differentiation, ASCs were cultured in medium containing 0.5 mM isobutylmethylxanthine, 60 mM indomethacin, 100 nM dexamethasone, and 10 mg/ml insulin (adipocyte differentiation medium). ASCs were grown in a 12-well plate with medium changes every 3 days. The presence of adipocytes was verified by staining for triglycerides with Oil Red O, an indicator of intracellular lipid accumulation, and adipocytes counted in four random microscopic fields from two wells per group. All chemical reagents used in ASC differentiation were purchased from Sigma-Aldrich (St. Louis, MO, http://www.sigmaaldrich.com).

### 2.4. ELISA

The cell supernatant level of OPN was determined using the ELISA kit (Westang) as described in the manufacturer's instructions. Concentrations were calculated using a standard curve generated by OPN standards included in the kit.

### 2.5. Real-Time PCR

The total RNA was isolated from cell pellets and subjected to reverse transcription using the FastQuant RT Kit (TIANGEN Biotech, Beijing, China, http://www.tiangen.com). Quantitative real-time PCR was measured with the FastStart Universal SYBR Green Master (Roche Diagnostics, Mannheim, Germany) using the StepOnePlus Real-Time PCR System (Applied Biosystems, Foster City, CA, USA). The total amount of mRNA was normalized to endogenous *β*-actin mRNA. The primer sequences used for the quantitative PCR are provided in Supporting Information Table S1.

### 2.6. Western Blotting Analysis

Total cell lysate was prepared as described before [23]. Proteins at the same amount were separated by SDS-PAGE and transferred onto PVDF membranes. After probing with primary and secondary antibodies, an antigen-antibody complex was visualized by the enhanced chemiluminescence reagent SuperSignal (Pierce). Antibodies used in western blotting analysis were provided in Additional file 4: Table S2.

### 2.7. RNA Interference

siRNA specific for mouse OPN, DNMT1, and control siRNA with scrambled sequences were purchased from GenePharma (Shanghai, China, http://www.genomeditech.com). ASCs were grown to 80% confluency and transfected with 5 *μ*l siRNA (40 pM) using the DharmaFECT transfection reagent following the manufacturer's instruction.

### 2.8. Lentivirus Construction and Infection of Human ASCs

We constructed lentiviral vectors encoding scrambled shRNA and short-hairpin RNAs (shRNAs) targeting OPN [24]. We added 1 : 1 culture medium and lentivirus to cultured human ASCs and added 8 mg/ml polybrene (4 *μ*l) (Sigma-Aldrich), and then changed medium 24 hours later. OPN expression in target cells was detected by real-time PCR and western blot analysis.

### 2.9. Adenovirus Vector Construction and Transfection

Overexpressed adenovirus specific for mouse OPN and control adenovirus vector were purchased from Genomeditech (Shanghai, China, http://www.genomeditech.com). Target cells were infected using 10^8^ PFU adenovirus. OPN expression in target cells was detected by real-time PCR.

### 2.10. Genomic DNA Extraction and Methylation-Specific PCR (MSP)

DNA was extracted from mouse ASCs using the TIANamp Genomic DNA Purification Kit (TIANGEN Biotech) according to the manufacturer's instructions. The same amount of genomic DNA was treated with sodium bisulfite as described (TIANGEN Biotech) and subjected to methylation-specific PCR (MS-PCR) analysis. Primers specific for methylated DNA were TIPMF (5′-TTTTATGGATTTTTGATGTTTTTTC-3′) and TIPMR (5′-CATAAAATTTTTACCACTACCCGAC-3′); primers for unmethylated DNA were TIPUF (5′-TTTATGGATTTTTGATGTTTTTTTG-3′) and TIPUR (5′-ATAAAATTTTTACCACTACCCAAC- 3′). The two regions of primers were designed to amplify the same region in the 5′-untranslated region (UTR) of OPN gene from -229 to -71. The PCR conditions were as follows: 95°C for 2 min, 38 cycles of 94°C for 30 s, 60°C for 30 s, and 72°C for 20 s and a final elongation at 72°C for 5 min. PCR products were electrophoresed by the ethidium bromide gels. DNA from control subjects was used as a negative control. Mouse methylated (P) and unmethylated (N) DNA standards were from A&D Technology. Results from duplicate experiments were used to determine the methylation status.

### 2.11. Evaluation of Glucose Homeostasis

Intraperitoneal glucose tolerance test (IPGTT) was carried out one week prior to the end of the experiments. Mice were injected with glucose at 2 g/kg body weight after an 8 h fast. Blood samples were taken at 0, 30, 60, and 120 min points, and glucose concentrations were determined using glucose meters from Nipro Diagnostics Inc. (Fort Lauderdale, FL).

### 2.12. Histochemical Analysis

Hematoxylin and eosin (H&E) staining was performed using tissue sections at a thickness of 6 *μ*m. We quantified adipocyte size by quantification analysis using an imaging system (ImageJ, National Institutes of Health).

### 2.13. Statistical Significance

The experiments were performed at least three times. Data were expressed as “mean ± SEM.” Differences between groups were analyzed by using Student's *t*-test associated with Excel software. Statistical significance was set to ^∗^ *P* < 0.05, ^∗∗^ *P* < 0.01, and ^∗∗∗^ *P* < 0.001.

## 3. Results

### 3.1. OPN Expression Was Reduced in Adipose Tissues and ASCs from Obese Mice or Overweight People

To assess the role of OPN in obesity, a mouse model of high-fat diet- (HFD-) induced obesity was established as described in Material and Methods. Control mice with normal chow diet (CD) showed an average rate of body weight gain at 0.7 g/week, while that of mice with high-fat diet was 1.3 g/week. Eventually, the difference in the body weight between the two groups was 7.45 g (Supplemental file 1: Figure S1A) which can be easily recognized by the naked eyes. Besides, we detected blood glucose levels in both groups and found that blood glucose was elevated in the high-fat diet group and reached diabetes standard (Supplemental file 1: Figure S1B). All these indicated that the obese mouse model was successfully constructed.

**Figure 1:**
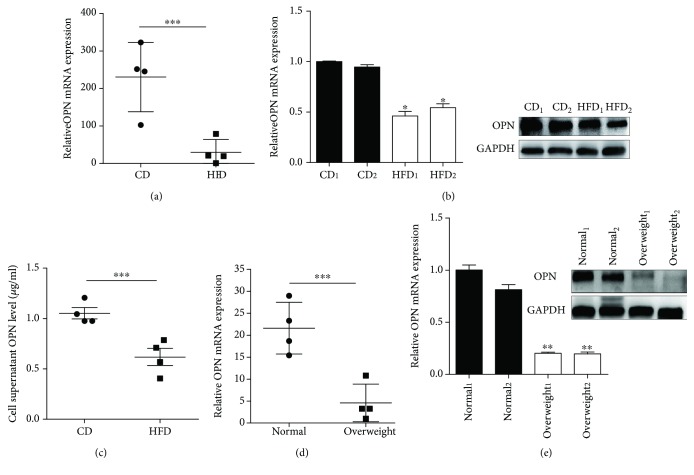
OPN (osteopontin) expression was detected in adipose tissue and ASCs (adipose-derived mesenchymal stem cells). (a) The expression of OPN mRNA in adipose tissue from high-fat diet- (HFD-) induced mice relative to the ones from chow diet- (CD-) induced mice. (b) Expression of OPN was detected by real-time PCR and western blot in ASCs from HFD and CD mice. (c) HFD ASCs and CD ASCs (1 × 10^6^ cells) were cultured in 2 ml complete medium for 48 hours. The supernatant was collected and centrifuged at 1000 × g for 4 minutes, and the OPN concentration was measured by ELISA. The OPN content of the adipose tissue (d) and ASCs in overweight and normal human subjects. (e) OPN expression was analyzed by real-time PCR and western blot. Values are means ± SEM. ^∗^ *P* < 0.05; ^∗∗^ *P* < 0.01; ^∗∗∗^ *P* < 0.001. All experiments were repeated at least thrice.

In adipose tissues from HFD mice, the expression of OPN was significantly lower than that of CD mice (Figure 1(a)). Considering the importance of adipose-derived mesenchymal stem cells (ASCs) in adipose tissues, we analyzed the expression of OPN in ASCs from HFD and CD mice. The results showed that OPN mRNA and secreted OPN protein in ASCs were significantly lower in HFD mice than in CD mice (Figures 1(b) and 1(c)). Further, we compared the OPN content in adipose tissues and ASCs from overweight and normal people, overweight defined as 25 ≤ BMI< 30 kg/m^2^ according to the World Health Organization (World Health Organization, 2000), and found similar results with that in mice (Figures 1(d) and 1(e)). These results demonstrated that the expression of OPN was decreased in adipose tissues and ASCs from both HFD mice and overweight people, suggesting an important role of OPN in obesity.

### 3.2. Methylation of OPN Promoter Inhibited the Transcription of OPN in HFD ASCs

**Figure 2:**
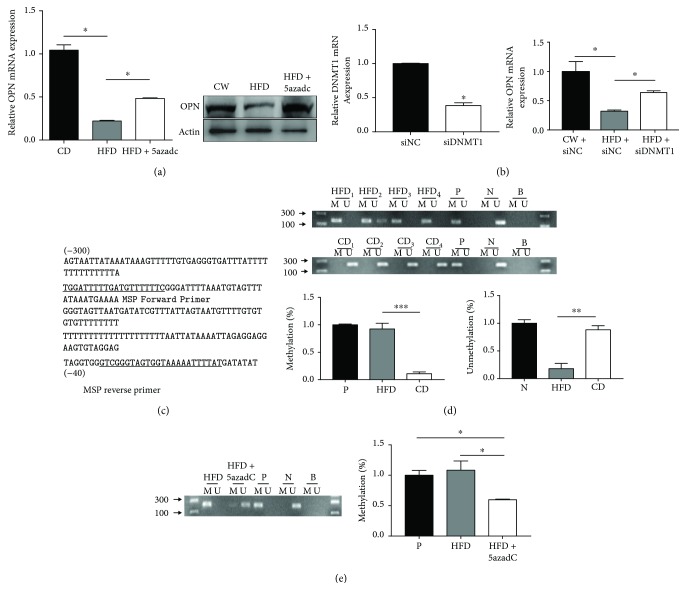
The methylation of the OPN promoter inhibits the transcription of OPN. (a) ASCs from HFD mice treated with 5 *μ*mol 5′-aza-2′dC (HFD + 5′-aza-2′dC) for 2 days related to untreated (HFD) and control (CD) cells was determined by real-time PCR and western blot analysis. (b) Effect of siDNMT1 treatment on the relative abundance of transcript levels of DNMT1 and OPN. (c) Relative location for primers of MS-PCR. (d) MS-PCR analysis of OPN in ASC lines from CD and HFD mice. Same amount of DNA was amplified with primers specific to the unmethylated (U) or the methylated (M) CpG sites of the OPN gene after modification with sodium bisulfite. Methylation or unmethylation percentage in ASCs have been quantitated by ImageJ (National Institutes of Health) with normalization to positive (P) or negative (N) control. (e) MS-PCR analyzed the methylation patterns of OPN promoters in ASCs after treatment with 5′-aza-2′dC for 2 days. The schematic showed the methylation percentage in response of 5′-aza-2′dC treatment. P, methylated DNA; N, unmethylated DNA; B, blank control. Values are means ± SEM. ^∗^ *P* < 0.05; ^∗∗^ *P* < 0.01; ^∗∗∗^ *P* < 0.001. All experiments were repeated at least thrice.

Recent studies suggest that DNA methylation-induced alteration of gene expression contributes to obesity [25, 26]. To explore whether decreased expression of OPN in ASCs from obese individuals is due to DNA methylation on the *OPN* promoter, HFD ASCs were treated with DNA demethylating agent 5′-aza-2′dC and siDNMT1, which was incorporated into DNA and caused an irreversible inactivation of DNA methyltransferase. OPN expression was significantly enhanced by the addition of 5′-aza-2′dC and siDNMT1 in HFD ASCs, suggesting that decreased OPN expression in HFD ASCs may be due to methylation of the *OPN* gene (Figures 2(a) and 2(b)).

The *OPN* promoter is not located within CpG islands according to the criteria established by the MethPrimer software (http://www.urogene.org/methprimer/index1.html), which defines a CpG island as showing >50% CG content and an observed/expected CpG frequency of >0.6. However, previous studies showed that genes with non-CpG island promoters share many epigenetic features that are associated with CpG island promoter genes, despite their low CpG density [27]. MS-PCR was performed to determine the methylation status of the OPN promoter. The CpG sites and primers specific for methylated and unmethylated DNA were analyzed by MethPrimer software (http://www.urogene.org/methprimer/index1.html) (Figure 2(c)). OPN promoters were partially methylated in ASCs from CD mice, which had abundant OPN mRNA expression. In contrast, OPN was highly methylated in HFD ASCs which had low OPN mRNA (Figure 2(d)). Besides, treatment with 5′-aza-2′dC decreased methylated MS-PCR products and increased unmethylated MS-PCR products in HFD ASCs (Figure 2(e)). Moreover, treatment of histone deacetylase (HDAC) inhibitor trichostatin A (TSA) greatly enhanced OPN expression in HFD ASCs (Supplemental file 2: Figure S2A), which indicates that histone deacetylation also contributes to the inactivation of OPN in HFD ASCs. These data demonstrate that methylation-induced epigenetic silence causes decreased OPN expression in obese adipose tissues.

### 3.3. OPN Silencing Promoted ASCs to Differentiate into Adipocytes

**Figure 3:**
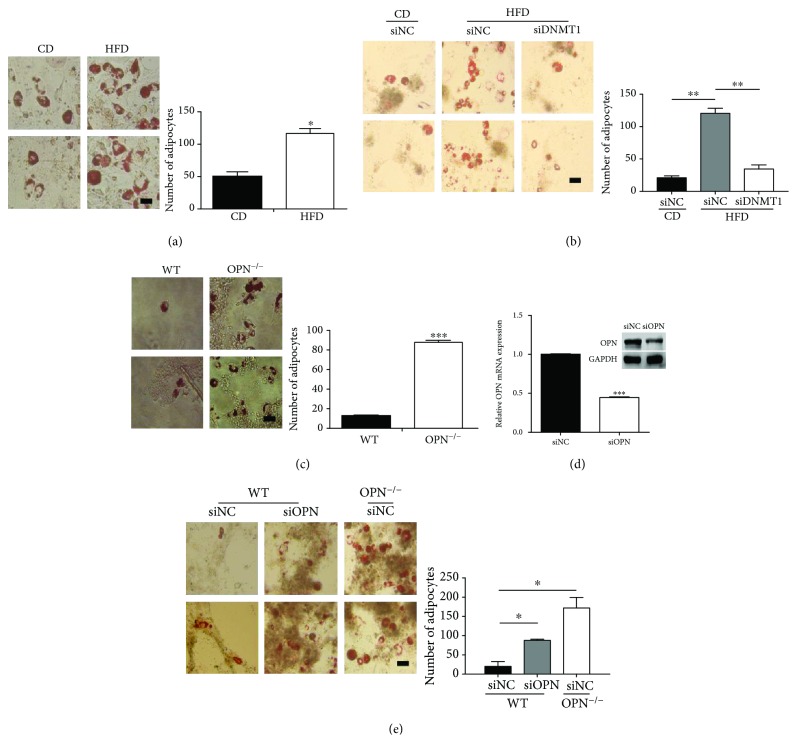
OPN deficiency accelerates ASC differentiation into adipocytes. (a) ASCs from CD mice or from HFD mice were cultured in adipogenic differentiation medium for 4 days and stained with Oil Red O to reveal lipid droplets. (b) CD and HFD ASCs, transfected with DNMT1 siRNA (siDNMT1) or control siRNA (siNC), were cultured in adipogenic differentiation medium for 3 days and stained with Oil Red O to reveal lipid droplets. (c) Wild-type or OPN^−/−^ ASCs were cultured in adipogenic differentiation medium for 3 days and stained with Oil Red O to reveal lipid droplets. (d) Wild-type ASCs transfected with OPN siRNA (siOPN) or control siRNA (siNC) were analyzed for OPN mRNA and protein 3 days after transfection, to verify knockdown efficiency. (e) Two groups of ASCs were subjected to adipogenic differentiation medium for 6 days and stained with Oil Red O. Adipocyte size was quantification analyzed by an imaging system (ImageJ, National Institutes of Health). (Bar = 50 *μ*m; adipocytes counted in four random microscopic fields from two wells per group.) Values are means ± SEM. ^∗^ *P* < 0.05; ^∗∗^ *P* < 0.01; ^∗∗∗^ *P* < 0.001. All experiments were repeated at least thrice.

We further determined the effect of OPN on ASC differentiation. The ASCs were cultured in adipogenic-induction medium for 4 days. Compared with CD ASCs, adipogenic differentiation was dramatically accelerated in HFD ASCs, which indicates that HFD ASCs have stronger adipogenic differentiation potential compared with cells from CD mice, according to the enumeration of adipocytes in four random microscopic fields for each group (Figure 3(a)). Moreover, knockdown of DNMT1 greatly attenuated adipogenic differentiation of HFD ASCs (Figure 3(b)), which further emphasized the importance of DNA methylation-induced OPN inactivation during adipogenic differentiation. A previous report has shown that OPN plays an important role in regulating the expression of adipogenic transcription factors [21]. To determine if OPN deficiency affects the adipogenic differentiation of ASCs, we isolated ASCs from wild-type (WT) mice and OPN^−/−^ mice. OPN expression was undetectable in OPN^−/−^ ASCs (Supplemental file 2: Figure S2). After being cultured in adipogenic-induction medium for 3 days, OPN^−/−^ ASCs show increased tendency for differentiation into adipocytes, compared with WT ASCs (Figure 3(c)). To confirm its role in the differentiation of ASCs, OPN was interfered in WT ASCs using siRNA. OPN expression was analyzed by real-time PCR and western blot (Figure 3(d)). Compared with wild-type ASCs, adipogenic differentiation was dramatically accelerated in OPN^−/−^ ASCs (Figure 3(e)). Thus, OPN is likely to play a key role in determining adipogenic differentiation of ASCs.

### 3.4. Extracellular OPN Inhibited ASC Adipogenic Differentiation through CD44- and Integrin *αν*/*β*1-Containing Receptors

**Figure 4:**
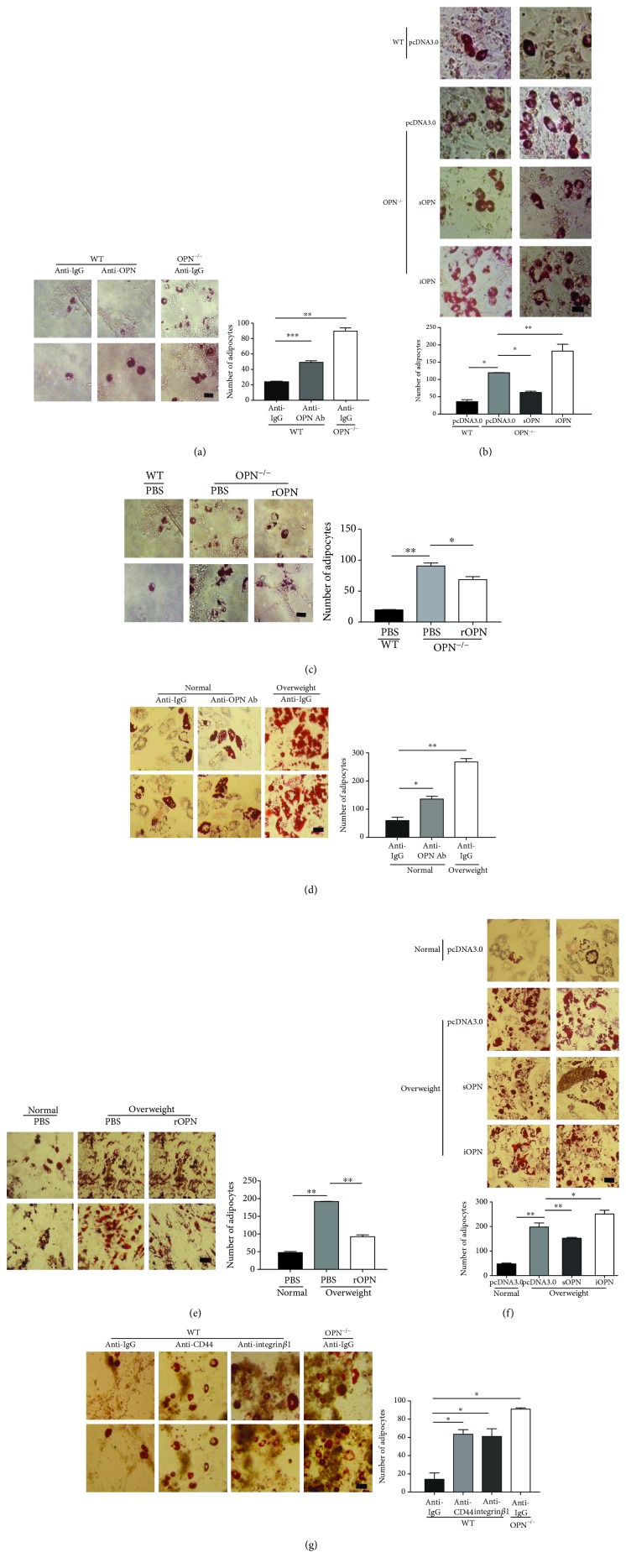
OPN retards the adipogenic differentiation of ASCs mainly in an extracellular manner. (a) WT and OPN^−/−^ ASCs were cultured in adipogenic differentiation medium supplemented with OPN-specific antibody (anti-OPN Ab) or isotype control (each 10 *μ*g/ml), then stained with Oil Red O after 4 days. (b) OPN^−/−^ and WT ASCs, transfected with secreted OPN (sOPN), intracellular OPN (iOPN), or pcDNA3.0, were cultured in adipogenic differentiation medium for 6 days, then stained with Oil Red O. (c) OPN^−/−^ and WT ASCs were cultured in adipogenic differentiation medium supplemented with recombinant OPN (rmOPN, 1 *μ*g/ml) or PBS [27] for 4 days and stained with Oil Red O. (d) hASCs, from overweight (25 ≤ BMI< 30) and normal (BMI < 25) human subjects, were cultured in adipogenic differentiation medium supplemented with OPN-specific antibody (anti-OPN Ab) or isotype control (each 10 *μ*g/ml) for 6 days, then stained with Oil Red O. (e) Overweight and normal ASCs were cultured in adipogenic differentiation medium exposed to PBS and recombinant OPN protein (each 1 *μ*g/ml) (rOPN) for 4 days, and then stained with Oil Red O. (f) Overweight and normal ASCs, transfected with secreted OPN (sOPN), intracellular OPN (iOPN), or pcDNA3.0, were subjected to adipogenic differentiation medium for 6 days, then stained with Oil Red O. (g) WT and OPN^−/−^ ASCs supplemented with blocking antibodies against integrin *αν*/*β*1 or CD44 or an isotype control (lgG; each 5 *μ*g/ml) was cultured in adipogenic differentiation medium for 4 days and stained with Oil Red O. Adipocyte size was quantification analyzed by an imaging system (ImageJ, National Institutes of Health). (Bar = 50 *μ*m; adipocytes counted in four random fields from two wells per group.) Values are means ± SEM. ^∗^ *P* < 0.05; ^∗∗^ *P* < 0.01; ^∗∗∗^ *P* < 0.001. All experiments were repeated at least thrice.

To further investigate the role of OPN in ASC differentiation, a specific monoclonal antibody was used to neutralize OPN *in vitro*. For WT ASCs, adipogenic differentiation was obviously augmented by the presence of an anti-OPN antibody (Figure 4(a)). As OPN exists in both secreted and intracellular isoforms, an antibody neutralization study suggests that secreted OPN plays an important role in regulating ASC differentiation. To confirm the result, sOPN (secreted OPN) and iOPN (intracellular OPN) plasmids were transfected into OPN^−/−^ ASCs. OPN expression was verified by real-time PCR (Supplemental file 3: Figure S3A). After treatments with adipogenic-induction medium, OPN^−/−^ ASCs with sOPN plasmid reduced adipogenesis, while iOPN plasmid could not reverse OPN deletion-induced adipogenesis and could even promote adipogenesis (Figure 4(b)). To verify the effect of extracellular OPN, we also reintroduced exogenous OPN during ASC differentiation. While OPN^−/−^ ASCs in adipogenic differentiation medium were supplemented with recombinant OPN protein, these ASCs weaken the capacity for adipogenic differentiation (Figure 4(c)).

Further, we found the same phenomenon by adding anti-OPN Ab to the supernatant of ASCs from a normal weight human and recombinant OPN protein to the supernatant of ASCs from an overweight human which confirmed more strongly that OPN works by extracellular forms (Figures 4(d) and 4(e)). In human subjects, by the secreted overexpression and intracellular OPN plasmid in overweight ASCs, OPN expression was verified by real-time PCR (Supplemental file 3: Figure S3B) and the same tendency can be observed (Figure 4(f)). The observation indicated that OPN could inhibit ASC differentiation through extracellular receptors.

Previous studies have shown that OPN works by binding to cell surface receptors [28], and integrin *αν*/*β*1 and CD44 are indeed highly expressed on MSCs [29]. We then used specific antibodies against the OPN receptors to determine which receptors mediate the effect of OPN on ASC differentiation. Treatment with antibody against integrin *β*1 led to enhanced adipogenesis treatment of ASCs with antibody against integrin *αν*/*β*1 which led to increased adipogenic capacity, mimicking the effect of OPN neutralizing antibodies, and anti-CD44 had similar effect (Figure 4(g)). These results strongly suggest that OPN regulates adipogenesis differentiation of ASCs initiated by binding to integrin *αν*/*β*1 and CD44.

### 3.5. OPN Exerts Its Effect on the Adipogenic Differentiation of ASCs via the C/EBP Pathway

**Figure 5:**
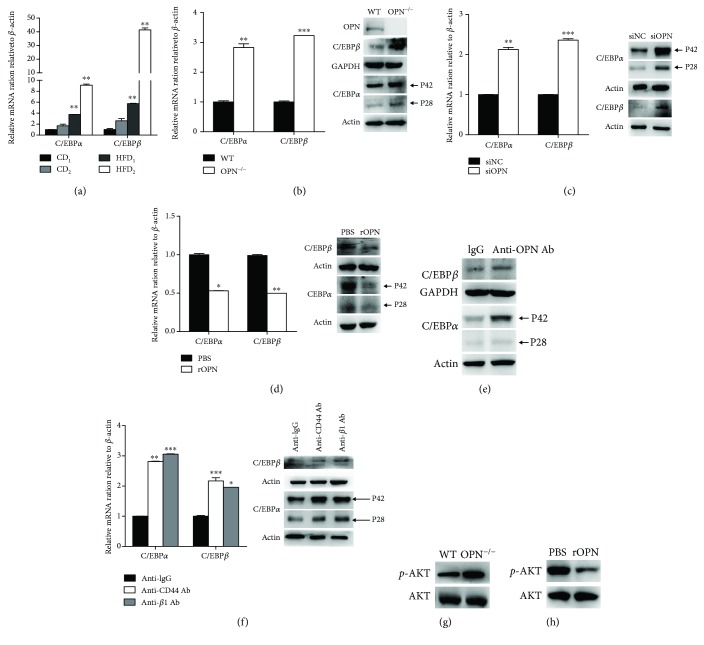
C/EBPs are involved in the OPN-directed adipogenic differentiation of ASCs. (a) Total RNA from CD or HFD ASCs was assayed for C/EBP*α* and C/EBP*β* mRNA by real-time polymerase chain reaction (PCR). (b) Total RNA from WT or OPN^−/−^ ASCs was assayed for C/EBP*α* and C/EBP*β* mRNA by real-time PCR. Total protein from OPN^−/−^ or WT ASCs was analyzed for OPN, C/EBP*α*, C/EBP*β* (b), and p-AKT (g) by western blotting. (c) Total RNA and protein preps from the WT ASCs transfected with OPN siRNA (siOPN) or control siRNA (siNC) assayed for C/EBP*α* and C/EBP*β* 3 days after transfection. (d) Total RNA and protein preps from OPN^−/−^ ASCs with rOPN (rOPN) or PBS were assayed for C/EBP*α* and C/EBP*β* by real-time PCR and western blot, and (h) protein preps from the same cells were assayed for p-AKT by western blot 2 days after addition. WT ASCs were supplemented with OPN-specific antibody (anti-OPN Ab) (10 *μ*g/ml) for 2 days. Total protein preps from WT ASCs with anti-OPN Ab (anti-OPN Ab) and control cells (lgG) were assayed for C/EBP*α* and C/EBPβ (e) by western blot. (f) Wild-type ASCs were supplemented with blocking antibodies against CD44 or integrin *αν*/*β*1 or an isotype control (each 5 *μ*g/ml) for 2 days. Total RNA and protein preps from WT ASCs with CD44 (anti-CD44) or integrin *αν*/*β*1 Ab (anti-*β*1 Ab) and control cells (anti-lgG) were assayed for C/EBP*α*, C/EBP*β* by real-time PCR and western blot. Values are means ± SEM. ^∗^ *P* < 0.05; ^∗∗^ *P* < 0.01; ^∗∗∗^ *P* < .001. All experiments were repeated at least thrice.

Studies have shown that C/EBPs, including C/EBP*β* and C/EBP*α* transcription factors, play a vital role to promote the expression of genes involved in adipogenic differentiation [17]. First, we compared the activation level of the signaling pathway in HFD and CD ASCs. It has been shown that there is greater mRNA expression for C/EBPs, key adipogenic differentiation factors, in HFD ASCs as compared to CD cells, which indicates that the signaling pathway does change (Figure 5(a)). In order to clarify the mechanism of OPN-regulating adipogenic differentiation of ASCs, we analyzed these important transcription factors in ASC differentiation. We found that the mRNA and protein expression of C/EBP*α* and C/EBP*β* were significantly increased in OPN^−/−^ ASCs compared to WT ASCs (Figure 5(b)). In WT ASCs treated with OPN-targeted small interfering RNA (siRNA), the similar expression was detected (Figure 5(c)). What is more, the C/EBPs have been shown to have the same trend by adding recombinant OPN protein to the OPN^−/−^ ASC supernatant (Figure 5(d)), anti-OPN Ab (Figure 5(e)), and blocking antibodies (Figure 5(f)) to the WT ASC supernatant.

It has been reported that the PI3K-AKT signaling pathway is involved in adipogenic differentiation of ASCs [30]. We also found that AKT phosphorylation was increased in OPN^−/−^ ASCs (Figure 5(g)), a finding that was verified by using OPN recombinant protein in OPN^−/−^ ASCs (Figure 5(h)).

In summary, these data suggest that OPN has an inhibitory effect on C/EBP transcription factors as well as adipogenesis and that OPN deficiency leads to upregulation of C/EBP transcription factors, favoring ASCs to adipose differentiation.

### 3.6. Effect of OPN on Body Weight of Obese Mice Induced by High-Fat Diet

**Figure 6:**
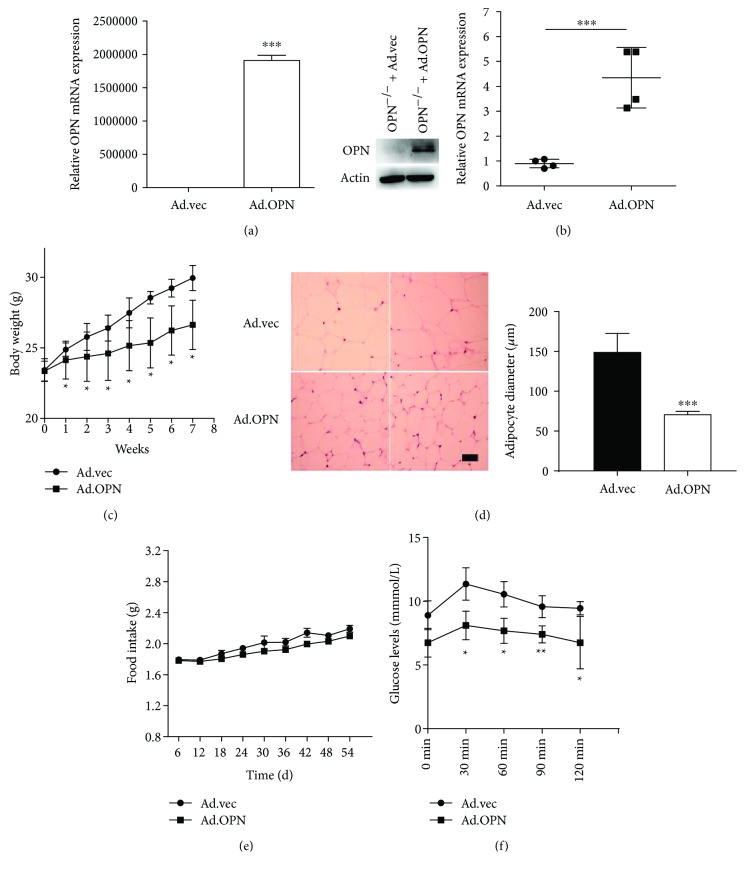
Effects of OPN on body weight of C57BL/6 obese mice induced by high-fat diet. (a) OPN^−/−^ ASCs transfected with OPN adenovirus (Ad.OPN) or control adenovirus vehicle (Ad.vec) were analyzed for OPN mRNA and protein for 3 days after transfection, to verify efficiency. (b) OPN mRNA level in ASCs from Ad.OPN and Ad.vec 8 weeks after OPN infection. (c) Weight change in the two groups of mice. (d) Representative images of H&E staining of subcutaneous adipose tissue from control Ad.vec mice and Ad.OPN mice (bar = 50 *μ*m), and the average diameter of adipocytes was quantification analyzed by ImageJ. (e) Food consumption was determined by measuring the difference between the amount provided and the amount left every three days. Food intake per mouse was calculated based on the amount consumed divided by time and the number of mice per cage. (f) Time-dependent blood concentration of glucose upon i.p. injection of glucose (1.5 g/kg) 8 weeks after OPN infection. Values are means ± SEM. ^∗^ *P* < 0.05; ^∗∗^ *P* < 0.01; ^∗∗∗^ *P* < 0.001.

To further investigate the role of OPN in the development of obesity, adenovirus was used as a viral delivery vehicle for *in vivo* studies. The infection efficiency of adenovirus expressing OPN (Ad.OPN) was examined *in vitro* after ASC infection with Ad.OPN or Ad.vec (control empty Ad) (Figure 6(a)). After treatment of high-fat diet with Ad.OPN or Ad.vec intravenous injection for 8 weeks, a significant increase of the OPN mRNA level was detected in ASCs isolated from the mice inguinal fat pads, which indicated that OPN gene expression can be successfully achieved (Figure 6(b)). We observed an obvious decrease in body weight after 8 weeks' injection in Ad.OPN mice compared to Ad.vec mice (Figure 6(c)). Animals injected with Ad.OPN eventually reached a steady state with an average body weight of 26.95 g by the end of the experiment, while Ad.vec mice reached an average of body weight of 29.7 g. Histological analysis of the adipose tissues showed the reduced sizes of individual adipocytes in the Ad.OPN group mice compared to those in the Ad.vec group, confirmed by quantification analysis using an imaging system (Figure 6(d)), which indicates that the OPN gene attenuates obesity by making the adipocytes smaller. In addition, injection of Ad.OPN did not cause either changes in food consumption of mice (Figure 6(e)) or apparent behavioral performance (data not shown). A glucose tolerance test revealed a lower peak level and much higher clearance rate of intravenous injected glucose in Ad.OPN mice (Figure 6(f)). Taken together, Ad.OPN into mice can delay the process of obesity and to some extent reduce blood glucose levels, which has a guiding significance for the treatment of obesity and its complications such as diabetes.

## 4. Discussion

Obesity is a worldwide health problem, and elucidation of the determinants of adipogenesis during the evolution of obesity is important to design targeted interventional strategies. Our studies observed that OPN expresses lower in ASCs and adipose tissue from overweight human beings and obese mice than in those from normal individuals. We also found that OPN is frequently inactivated due to methylation in ASCs and adipose tissue from obese mice. Furthermore, *in vitro* experiments showed that OPN inhibits adipogenic differentiation of ASCs by binding to extracellular receptors integrin *αν*/*β*1 and CD44. OPN adenovirus intravenous injection in mice to reduce obesity more effectively verified that the OPN might be a novel therapeutic target for attenuating obesity.

DNA methylation plays an important role in obesity and related metabolic complications [31]. Methylation of several gene promoters, such as HAND2, HOXC6, and PPARG, influences adipogenesis and differentiation, obesity, lipid metabolism, and adipose tissue expandability [26]. Consistent with these previous studies, we found that there is increased methylation in the OPN promoter, which inhibits OPN expression in adipose tissue and ASCs in obese mice, facilitating adipogenic differentiation of ASCs and adipogenesis. Moreover, treatment of TSA greatly enhanced OPN expression in HFD ASCs indicating that histone deacetylation also contributed to the inactivation of OPN in HFD ASCs. Histone modification-caused OPN inactivation in obese adipose tissues and ASCs still needs further investigation.

Controversially, previous studies have shown that mice lacking the *OPN* gene are protected by obesity and insulin resistance induced by a 24-week HF diet [32], which is due to a long-term HF diet that alters the microenvironment of mice, which results in a significant increase in various proinflammatory cytokines and immune cells in the fat tissue [33]. Additionally, aging may also affect fat accumulation as 24-week mice are older than 8-week ones [34]. What is more, mice lacking OPN were found to develop a higher fat-to-body weight ratio [21], which further proves that OPN, under different conditions, plays different roles in the control of fat deposition. Therefore, we believe that OPN affects the adipogenic process during early obesity.

As we know, OPN is an important factor for maintaining the stemness of the blood stem cells. In our previous study, we demonstrated that autocrine OPN is required for hepatic progenitor cells to maintain their stem-like properties. In addition, OPN promotes a cancer stem cell-like phenotype in hepatocellular carcinoma. In quiescent cancer stem cells, the expression of OPN is higher to maintain its self-renewal capacity. As we know, adipose-derived mesenchymal stem cells, with immunomodulatory properties and multilineage differentiation potential, become a great alternative source of acquiring stem cells, due to high abundance and a much safer and less time-consuming liposuction procedure [35]. In this study, we found that OPN can inhibit ASC adipogenic differentiation, which indicates that OPN might be important for ASCs maintaining their undifferentiated state. Thus, OPN could be used on ASC culturing and applied to address a wide range of disease conditions.

## 5. Conclusion

In summary, our study revealed that OPN expresses lower in adipose tissue and ASCs from obese mice because of methylation, eliciting the role of OPN in obesity. We showed that OPN could inhibit adipogenesis through inhibition of the C/EBP signal via integrin *αν*/*β*1 and CD44 and further attenuate obesity.

## Supplementary Materials

Supplementary Materials Figure S1: the construction of obese mice model. Figure S2: the expression of OPN mRNA by using HDAC inhibitor TSA was analyzed by RT-PCR. Figure S3: OPN expression determined by real-time PCR and western blotting analysis. Figure S4: OPN levels in ASCs transfected with secreted OPN (sOPN) and intracellular OPN (iOPN). Table S1: primer sequences used in this study. Table S2: primary antibodies used in this study.
