# Supplementary material for: Obesity-Induced Methylation of Osteopontin Contributes to Adipogenic Differentiation of Adipose-Derived Mesenchymal Stem Cells
Source: Stem Cells Int. 2019 Feb 17;2019:1238153. doi: 10.1155/2019/1238153 (PMC6398038; doi:10.1155/2019/1238153)
Supplement: Supplementary Materials — Figure S1: the construction of obese mice model. Figure S2: the expression of OPN mRNA by using HDAC inhibitor TSA was analyzed by RT-PCR. Figure S3: OPN expression determined by real-time PCR and western blotting analysis. Figure S4: OPN levels in ASCs transfected with secreted OPN (sOPN) and intracellular OPN (iOPN). Table S1: primer sequences used in this study. Table S2: primary antibodies used in this study. [file 1238153.f1.zip › 1238153.f1/Supplemental Figure S 1.doc]

**Figure S 1. The construction of obese mice model.** (**A**): Weight change of obese mice induced by high fat diet (HFD) and control mice (CD) in different times. (**B**) The mean blood glucose level of the two groups after 12 weeks diets. Values are means ± SEM, *, *P* < .05; **, *P* < .01; ***, *P* < .001. All experiments were repeated at least thrice.
