# Supplementary material for: Obesity-Induced Methylation of Osteopontin Contributes to Adipogenic Differentiation of Adipose-Derived Mesenchymal Stem Cells
Source: Stem Cells Int. 2019 Feb 17;2019:1238153. doi: 10.1155/2019/1238153 (PMC6398038; doi:10.1155/2019/1238153)
Supplement: Supplementary Materials — Figure S1: the construction of obese mice model. Figure S2: the expression of OPN mRNA by using HDAC inhibitor TSA was analyzed by RT-PCR. Figure S3: OPN expression determined by real-time PCR and western blotting analysis. Figure S4: OPN levels in ASCs transfected with secreted OPN (sOPN) and intracellular OPN (iOPN). Table S1: primer sequences used in this study. Table S2: primary antibodies used in this study. [file 1238153.f1.zip › 1238153.f1/Supplemental Figure S 4.doc]

**Figure S 4：OPN levels in ASCs transfected with secreted OPN (sOPN) and intracellular OPN (iOPN). (A):** OPN^-/-^ and WT ASCs were transfected with secreted OPN (sOPN) , intracellular OPN (iOPN), or pcDNA3.0 and OPN expression was determined by real-time PCR. **(B):** Overweight and normal ASCs were transfected with secreted OPN (sOPN) , intracellular OPN (i-OPN), or pcDNA3.0 and OPN expression was analyzed by real-time PCR.
