# Supplementary material for: Obesity-Induced Methylation of Osteopontin Contributes to Adipogenic Differentiation of Adipose-Derived Mesenchymal Stem Cells
Source: Stem Cells Int. 2019 Feb 17;2019:1238153. doi: 10.1155/2019/1238153 (PMC6398038; doi:10.1155/2019/1238153)
Supplement: Supplementary Materials — Figure S1: the construction of obese mice model. Figure S2: the expression of OPN mRNA by using HDAC inhibitor TSA was analyzed by RT-PCR. Figure S3: OPN expression determined by real-time PCR and western blotting analysis. Figure S4: OPN levels in ASCs transfected with secreted OPN (sOPN) and intracellular OPN (iOPN). Table S1: primer sequences used in this study. Table S2: primary antibodies used in this study. [file 1238153.f1.zip › 1238153.f1/Supplemental Table S 1.doc]

Table S 1. Primer sequences used in this study.

| **Primers for Real-time PCR** | |
| --- | --- |
| **Protein** | **Sequence (5’→3’)** |
| Mouse OPN forward | 5’- AGCAAGAAACTCTTCCAAGCAA -3’ |
| Mouse OPN reverse | 5’- GTGAGATTCGTCAGATTCATCCG -3’ |
| C/EBPα forward | 5’- CAAGAACAGCAACGAGTACCG -3’ |
| C/EBPα reverse | 5’- GTCACTGGTCAACTCCAGCAC -3’ |
| C/EBPβ foward | 5’-GCGGGAACGCAACAACATC-3’ |
| C/EBPβ reverse | 5’-GTCACTGGTCAACTCCAGCAC-3’ |
| Mouse β-actin forward | TTCCAGCCTTCCTTCTTGGG -3’ |
| Mouse β-actin reverse | TGTTGGCATAGAGGTCTTTACGG -3’ |
| Human β-actin forward | CATGTACGTTGCTATCCAGGC |
| Human β-actin reverse | CTCCTTAATGTCACGCACGAT |
| Human OPN forward | GAAGTTTCGCAGACCTGACAT |
| Human OPN reverse | GTATGCACCATTCAACTCCTCG |
| DNMT1 foward | CTGAGAAGCCCAAGGTCAAG |
| DNMT1 reverse | CAGCAGATGGTGCAGTAGGA |
