# Supplementary material for: Obesity-Induced Methylation of Osteopontin Contributes to Adipogenic Differentiation of Adipose-Derived Mesenchymal Stem Cells
Source: Stem Cells Int. 2019 Feb 17;2019:1238153. doi: 10.1155/2019/1238153 (PMC6398038; doi:10.1155/2019/1238153)
Supplement: Supplementary Materials — Figure S1: the construction of obese mice model. Figure S2: the expression of OPN mRNA by using HDAC inhibitor TSA was analyzed by RT-PCR. Figure S3: OPN expression determined by real-time PCR and western blotting analysis. Figure S4: OPN levels in ASCs transfected with secreted OPN (sOPN) and intracellular OPN (iOPN). Table S1: primer sequences used in this study. Table S2: primary antibodies used in this study. [file 1238153.f1.zip › 1238153.f1/Supplemental Table S 2.doc]

Table S 2. Primary antibodies used in this study.

| **Antibodies used in the research** | | |
| --- | --- | --- |
| **Protein** | **Usage** | **Antibody** |
| Mouse β-actin | WB | 3700 Cell signaling technology |
| Human β-actin | WB | 4970 Cell signaling technology |
| Mouse OPN | WB | 8448 Abcam |
| Human OPN | WB | 8448 Abcam |
| C/EBPα | WB | 8178S Cell signaling technology |
| C/EBPβ | WB | 3082S Cell signaling technology |
| Anti-OPN Ab | WB | 3688 Abcam |
| CD44 | Antibody neutralization experiment | 558739 BD |
| Interginα ν/β_1_ | Antibody neutralization experiment | 550024 BD |

Abbreviations: WB, Western blotting
